# Elastic modulus of β-Ga_2_O_3_ nanowires measured by resonance and three-point bending techniques

**DOI:** 10.3762/bjnano.15.58

**Published:** 2024-06-18

**Authors:** Annamarija Trausa, Sven Oras, Sergei Vlassov, Mikk Antsov, Tauno Tiirats, Andreas Kyritsakis, Boris Polyakov, Edgars Butanovs

**Affiliations:** 1 Institute of Solid State Physics, University of Latvia, LV-1063 Riga, Latvia; 2 Institute of Physics, University of Tartu, W. Ostwaldi 1, 50411 Tartu, Estonia; 3 Institute of Technology, University of Tartu, Nooruse 1, 50411 Tartu, Estonia; 4 Estonian Military Academy, Riia 12, 51010 Tartu, Estonia

**Keywords:** atomic force microscopy, elastic modulus, gallium oxide, mechanical properties, nanowire, scanning electron microscopy

## Abstract

Due to the recent interest in ultrawide bandgap β-Ga_2_O_3_ thin films and nanostructures for various electronics and UV device applications, it is important to understand the mechanical properties of Ga_2_O_3_ nanowires (NWs). In this work, we investigated the elastic modulus of individual β-Ga_2_O_3_ NWs using two distinct techniques – in-situ scanning electron microscopy resonance and three-point bending in atomic force microscopy. The structural and morphological properties of the synthesised NWs were investigated using X-ray diffraction, transmission and scanning electron microscopies. The resonance tests yielded the mean elastic modulus of 34.5 GPa, while 75.8 GPa mean value was obtained via three-point bending. The measured elastic moduli values indicate the need for finely controllable β-Ga_2_O_3_ NW synthesis methods and detailed post-examination of their mechanical properties before considering their application in future nanoscale devices.

## Introduction

Significant advancements in both material and device technologies related to monoclinic gallium oxide (β-Ga_2_O_3_) have been achieved in the current decade [1–2]. Recently, attention has been directed towards it due to its outstanding properties [3] such as ultrawide band gap (4.4–4.9 eV) and chemical stability [4–5]. Ga_2_O_3_ is a promising candidate for visible-blind UV-light sensors [3], power devices and optoelectronics [6–9], gas sensors [10], and memory devices [8].

These applications can be scaled down to the nanoscale, including flexible nanodevices. Ga_2_O_3_ nanowires (NWs) could be suitable for use on bendable and stretchable substrates in line with the current trends in electronic technologies focusing on flexible electronic device development [11–12]. Consequently, understanding the mechanical properties of β-Ga_2_O_3_ NWs becomes an important step. For instance, precise determination of the elastic modulus is essential for designing Ga_2_O_3_-based nanomechanical resonators or flexible field-effect transistors [13]. However, a minimal amount of research has been dedicated to exploring and understanding the mechanical properties of Ga_2_O_3_ NWs [14].

There are several methods available for studying the mechanical properties of NWs, such as nanoindentation [15], three-point bending tests using an atomic force microscope (AFM) [16], and in-situ scanning electron microscope (SEM) resonance [17]. However, challenges of obtaining consistent and comparable elastic modulus values across these different methods arise from multiple factors. For instance, different NW growth mechanisms and sensitive synthesis conditions, their structural and geometrical variations, beam theory model validity, and the resolution of microscopy techniques leading to inaccurate measurements of the NW dimensions, particularly at the lower resolution limit [18].

The low symmetry of monoclinic crystal systems, as in the β-Ga_2_O_3_ case, might promote the growth of nanostructures with different crystalline orientations, which often leads to the formation of nanostructures with various dimensions, such as NWs, nanobelts (NBs), and nanosheets/nanoplates [19–20]. Therefore, it is essential to understand how the elastic modulus values of NWs and NBs may differ. For instance, materials such as ZnO have demonstrated varying elastic modulus values depending on their geometrical dimensions [21–22]. Additionally, studies by Luan et al. [23] have revealed the elastic anisotropy in β-Ga_2_O_3_, highlighting the strong directional dependence of Young’s modulus. Available studies hint that various factors could strongly influence the mechanical properties of Ga_2_O_3_ one-dimensional nanostructures, which merits to be investigated more deeply.

This work addresses the challenges associated with determining the elastic modulus of individual monoclinic Ga_2_O_3_ NWs. Here, the selection and use of two distinct measurement techniques, resonance and three-point bending, was driven by the fact that the test method contributes to variations in reported nanostructure moduli. The mean elastic modulus of 34.5 and 75.8 GPa were obtained from resonance and three-point bending methods, respectively, which is significantly lower than the bulk value. Furthermore, the measured elastic moduli values indicate the need for finely controllable β-Ga_2_O_3_ NW synthesis methods and detailed post-examination of their mechanical properties before considering their application in future nanoscale devices.

## Results

**Figure 1:**
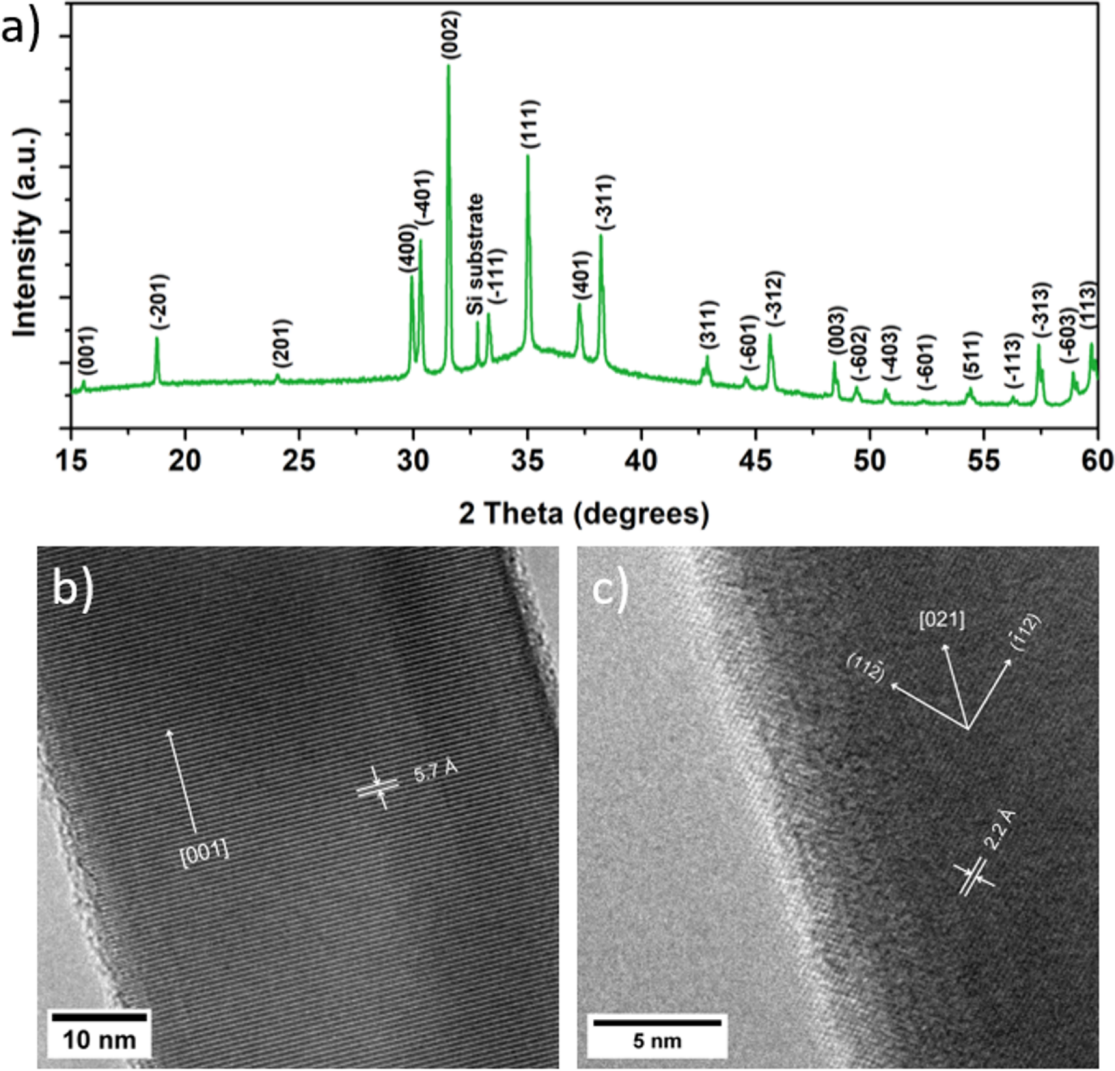
a) X-ray diffraction pattern of β-Ga_2_O_3_ NWs on silicon substrate. b) TEM image of a β-Ga_2_O_3_ NW with interlayer spacing of 5.7 Å, indicating [001] growth direction. c) TEM image of a β-Ga_2_O_3_ NW with orthogonal (11−2) and (−112) planes and interlayer spacing 2.2 Å, indicating [021] growth direction.

For structural analysis of the as-grown NW arrays on Si(100)/SiO_2_ substrates, X-ray diffraction (XRD) measurements were conducted. The marked peaks are associated with monoclinic β-Ga_2_O_3_ (ICDD-PDF #41–1103), as indicated in Figure 1a, while the Bragg peak at around 33 degrees corresponds to the Si substrate (forbidden Si(200) reflection). Furthermore, transmission electron microscopy (TEM) was used to study the inner crystalline structure of individual NWs (see Figure 1b and Figure 1c). The as-grown NWs typically are single crystalline without any distinguishable planar structural defects, such as twin boundaries or stacking faults. Fast Fourier transformation (FFT) was performed on the TEM images to ascribe the crystalline planes and determine the growth direction of the NWs. Several NWs were studied, and more than one growth direction was identified, which is common for β-Ga_2_O_3_ NWs [20]. For example, Figure 1b shows a NW with interlayer spacing of 5.7 Å indicating [001] growth direction, while in another NW, shown in Figure 1c, orthogonal (11−2) and (−112) planes were identified with 2.2 Å interlayer spacing, indicating [021] growth direction. The presence of NWs with various crystalline orientations suggests either the absence of one dominant preferential growth direction due to the low-symmetry monoclinic phase or other growth mechanisms alongside the vapour–liquid–solid (VLS) method.

**Figure 2:**
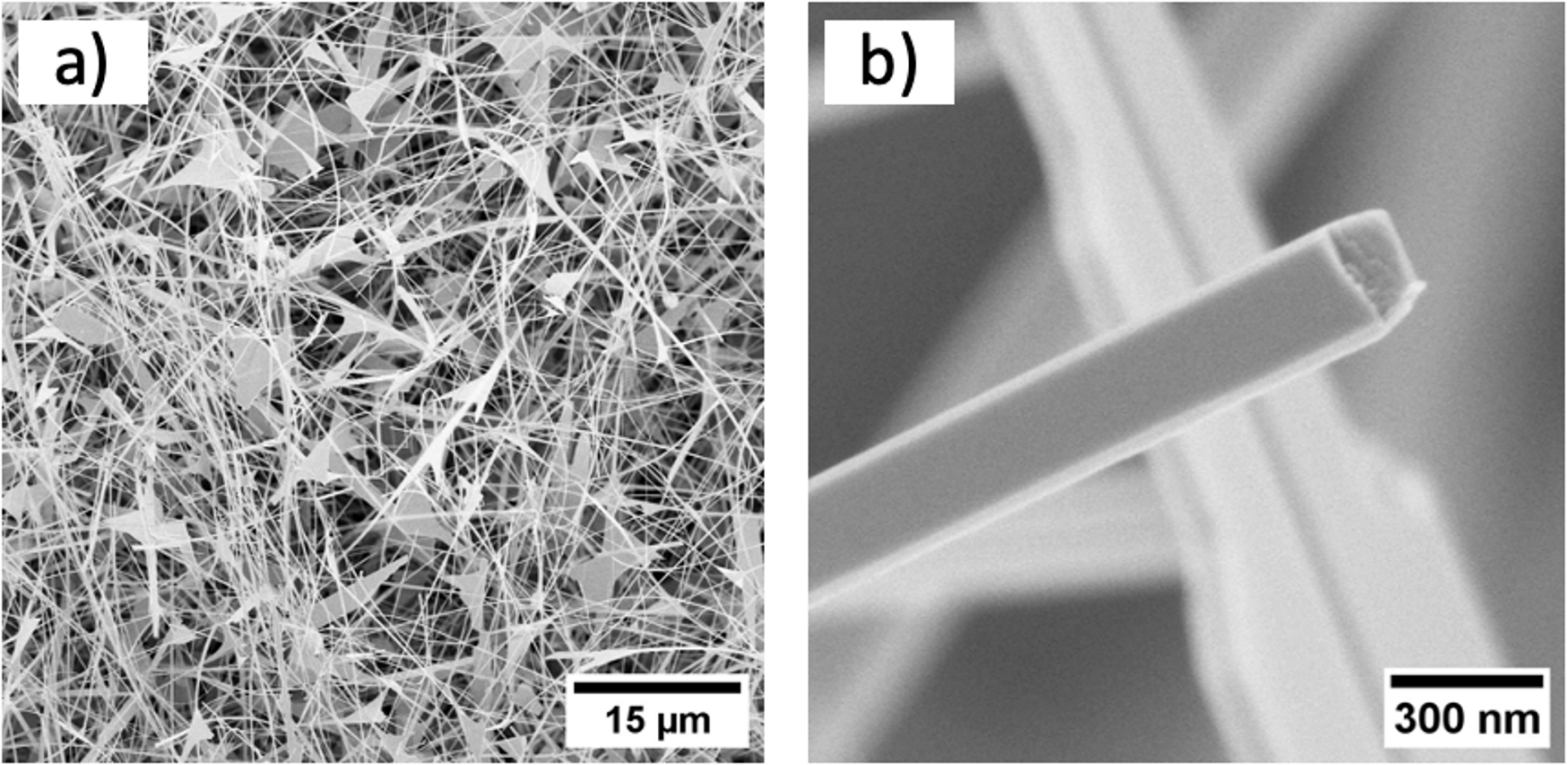
a) SEM image of the as-grown Ga_2_O_3_ NWs on silicon substrate; b) an individual NW with trapezoid cross-section.

Following an examination via SEM, it became evident that the NWs exhibit variations in their dimensions, highlighting a nonuniform geometry (Figure 2). The observed NWs and NB-like structures exhibited various cross-sections, including square-like, trapezoid, and rectangular shapes (see Figure S1 in Supporting Information File 1). Only part of the NWs had Au catalyst particles at the end of the NW, which is an indication of VLS growth; therefore, suggesting that a large portion of NWs grew via the self-catalytic vapour–solid (VS) mechanism [24]. Different growth mechanisms could potentially lead to variations in the NW structural properties, as was also indicated by the TEM study.

**Figure 3:**
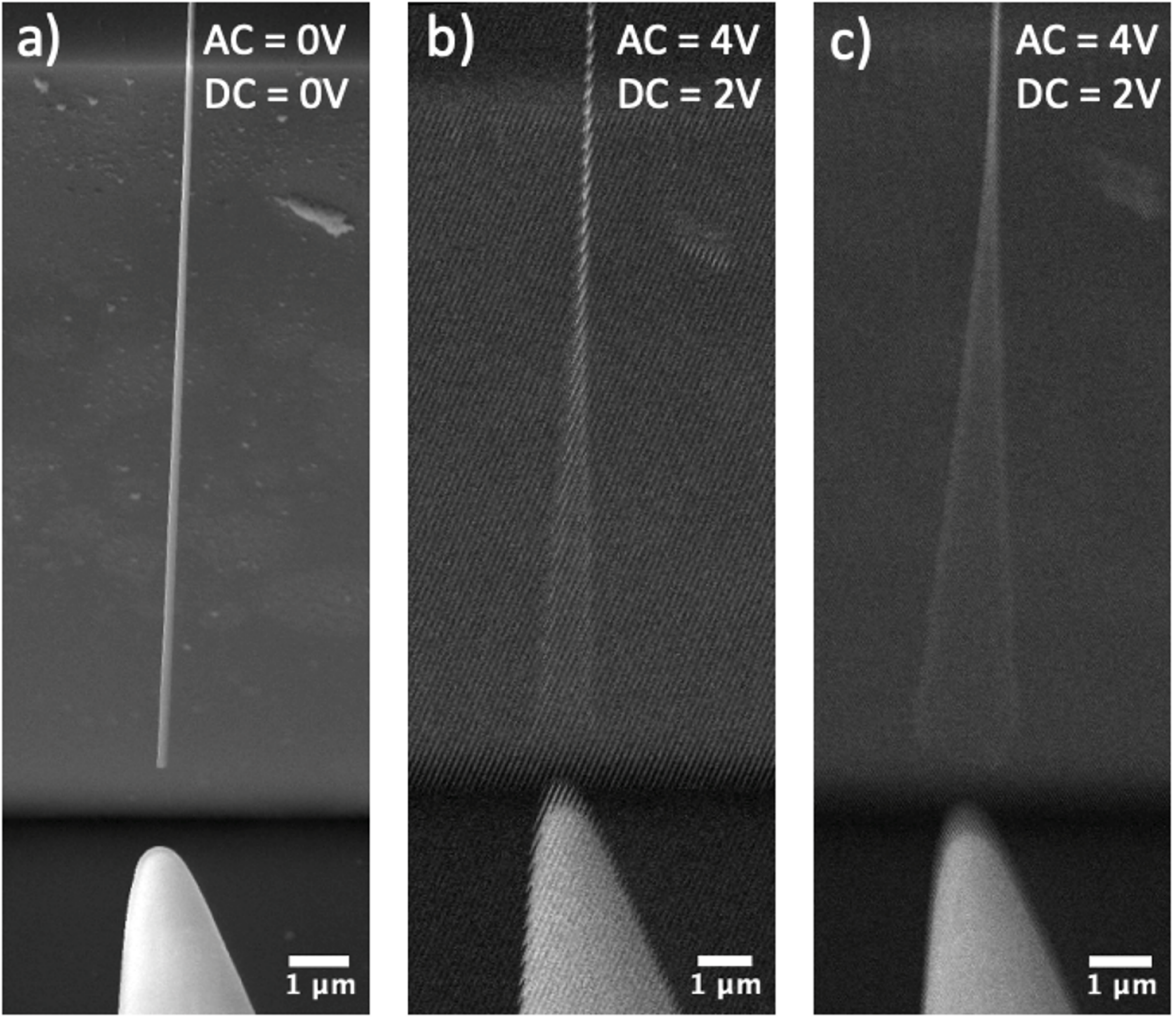
SEM image of a NW fixed at one end and a closely positioned probe tip: a) without applied AC and DC; b) observable oscillation with 4V AC and 2V DC (no resonance); c) with 4V AC and 2V DC (resonance).

**Figure 4:**
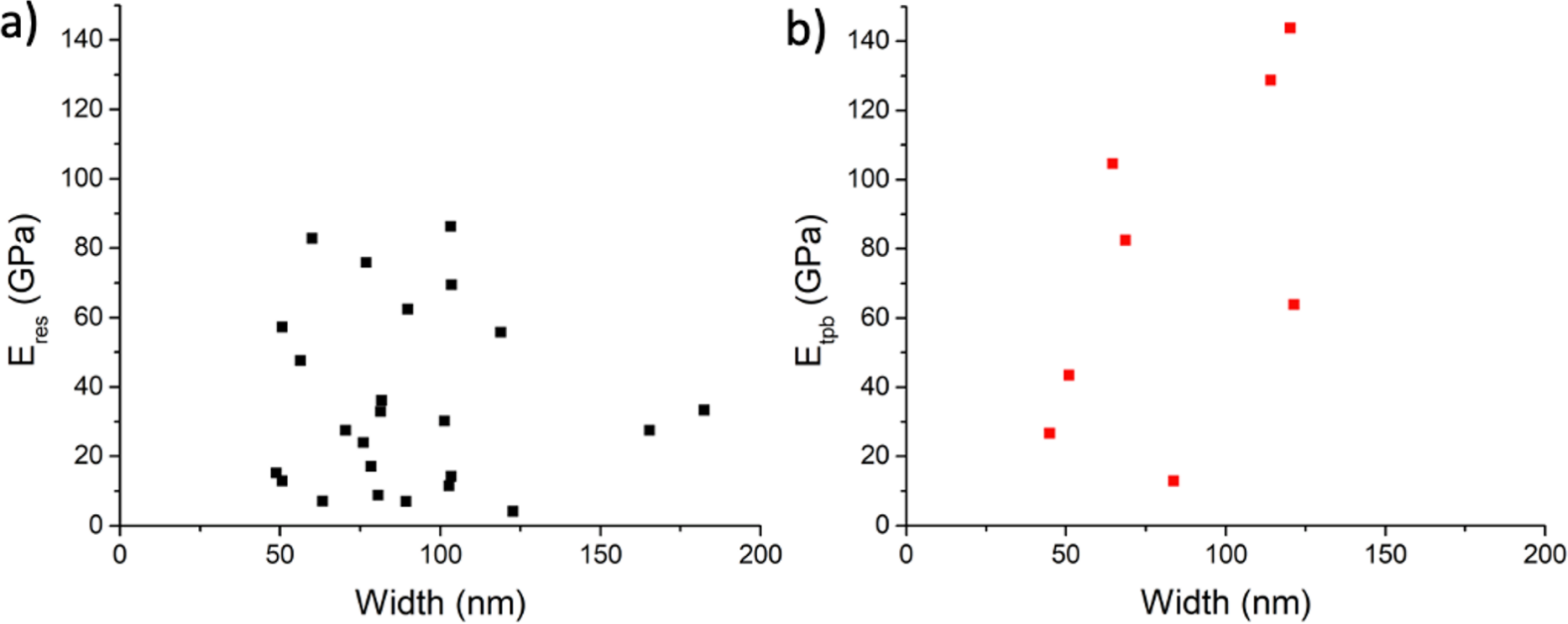
Elastic modulus plotted with respect to the width of Ga_2_O_3_ NWs: a) obtained via SEM resonance tests; b) obtained via AFM three-point bending tests.

The mechanical characteristics of Ga_2_O_3_ NWs were first examined using in-situ SEM resonance tests. Figure 3a–c presents a series of SEM images depicting an excitation of the first mode mechanical resonance in a Ga_2_O_3_ NW, characterised by a length *L* = 13.23 μm and width *w* = 102.79 nm with a resonance frequency *f*_1_ = 126.75 kHz. In the presence of a 4V AC voltage applied to the probe, the NW exhibits noticeable oscillations (Figure 3b). When the generated frequency aligns with the natural resonance frequency of the NW (Figure 3c), there is a substantial increase in the amplitude of NW oscillations. To ensure the fundamental natural resonance frequency has been correctly identified, instead of a parametric or forced resonance [25], oscillations at half of the resonance frequency were checked for each NW. In total, the resonance frequency of 26 NWs was measured (see Table S1 in Supporting Information File 1). The width of the NWs varied from 48 to 183 nm, while the length ranged from 6 to 27 µm. In the horizontal (y-axis, see the chosen coordinate system in Figure 6a) resonance oscillations measurements, only the width of the NW is measured for the elastic modulus calculation. The lack of sufficiently precise height measurement at both ends of the NW in this setup does not allow accurate elastic modulus value estimation from resonance oscillation in the vertical (x-axis) direction. The mean value of the elastic modulus was *E*_res_ = 34.5 GPa, which is significantly smaller than the reported theoretical Young's modulus for the bulk material (discussed in detail in the Discussion section) [26]. No dependence of the measured elastic modulus values on the geometrical dimensions of Ga_2_O_3_ NW was observed (see Figure 4a and Table S1 from Supporting Information File 1). The histogram of obtained elastic modulus values is shown in Figure S3 in Supporting Information File 1.

**Figure 5:**
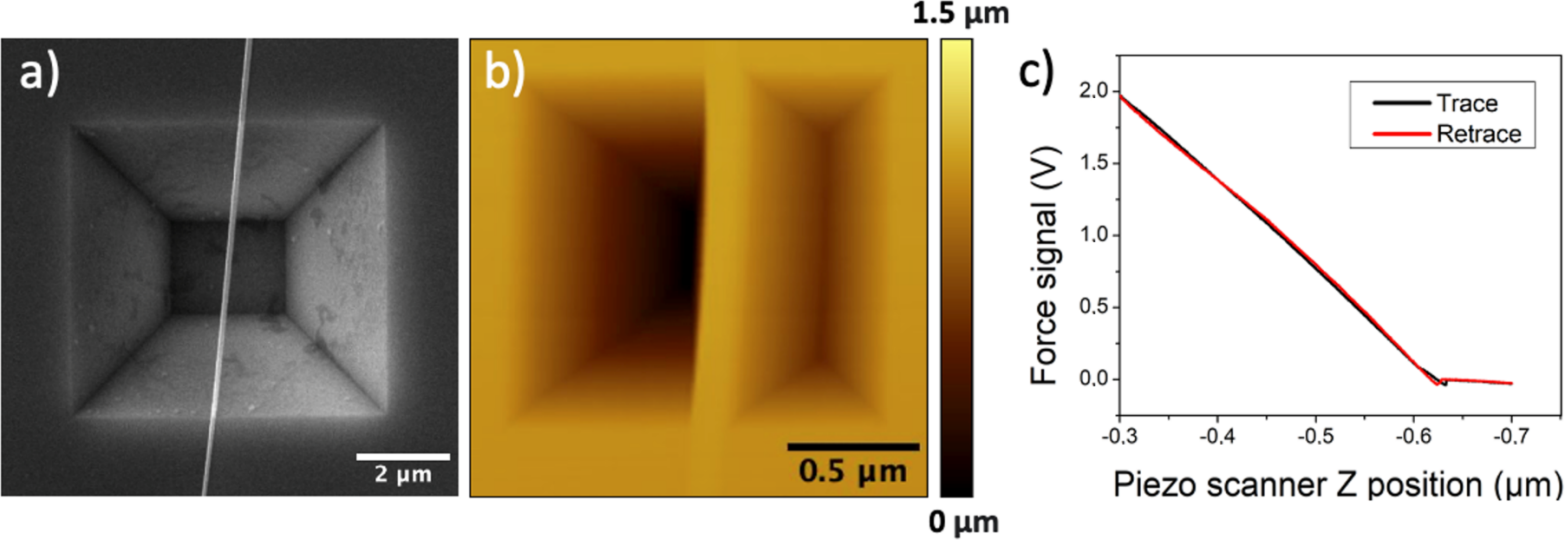
a) SEM image of Ga_2_O_3_ NW suspended over the inverted pyramid with both ends fixed. b) AFM topography image of Ga_2_O_3_ NW suspended over the inverted pyramid with both ends fixed, with the NW height determined to be approximately 23 nm. c) Loading and unloading spectra, illustrating one instance of three-point bending test.

The results of the three-point bending tests are shown in Figure 4b and in Table S3 in Supporting Information File 1, exhibiting significant data scattering without clear dependence on the geometrical dimensions, similar to the results of the resonance technique. The mean value of the elastic modulus was calculated to be *E*_tpb_ = 75.8 GPa, which is below the elastic modulus for bulk Ga_2_O_3_. While the length and width of NWs for three-point bending were measured in SEM, the heights were taken from the topography data obtained by AFM in the adhered parts of the NW at each end. In Figure 5a, an SEM image captures the morphology of a Ga_2_O_3_ NW positioned over an inverted pyramid structure. Notably, both ends of the NW are fixed, laying the foundation for a controlled three-point bending experiment. Figure 5b presents the AFM topography of the Ga_2_O_3_ NW. The loading and unloading spectra, illustrating one instance of the three-point bending test, is shown in Figure 5c. Since the elastic modulus depends on the height of the NW in the third power, errors in measuring the height can cause significant scattering. Furthermore, few NWs exhibited a nonuniform height distribution. For example, the height of the specific NW was slightly above 20 nm at one end but increased to 80 nm at the other end (see Figure S2 in Supporting Information File 1), leading to deviation from beam theory and giving inaccurate calculation of the elastic modulus in such case.

## Discussion

Observing differences in the elastic properties of our Ga_2_O_3_ NWs in comparison to bulk material, as well as a significant data scatter, prompted various potential explanations for this variation. Firstly, both experimental and theoretical investigations have shown that β-Ga_2_O_3_ elastic characteristics exhibit significant anisotropy [13,26–27]. This indicates that Young's modulus is strongly dependent on the direction of crystalline orientation (e.g., *E*_100_ = 138 GPa, *E*_010_ = 263 GPa, *E*_001_ = 228 GPa [26]) emphasising directional differences in the mechanical properties of the material. Since our as-grown NWs tend to exhibit various growth directions, as was determined in the TEM studies, this elasticity anisotropy could be one of the reasons for the scattering of the measured elastic modulus values.

Secondly, since the elastic modulus depends so intricately on the dimensions of the nanostructure (height is raised to the third power for three-point bending, width to the second power, and length to the fourth power for resonance frequency calculations), inaccuracies in measuring these dimensions can cause a scattering effect on the elastic modulus [28]. In this work, this effect is further complicated by the fact that β-Ga_2_O_3_ NW cross-sections tend to deviate from the rectangular geometry (e.g., trapezoid), giving an additional error to the calculations which should be minimised via a thorough study of each individual nanostructure geometry [29]. The magnitude of the geometrical measurement error typically increases significantly for smaller NWs, since the relative measurement error is becoming comparable to the absolute value of the dimension, leading to overestimation of the elastic modulus, which can be mistaken for the “size effect” [18]. The onset diameter of this size effect, when the surface contribution towards the NW stiffening is becoming significant, has been conflictingly reported to be around 10–40 nm and below [18,30]. In this work, most of the NWs had width values above 50 nm; therefore, elastic moduli variations from the bulk value due to surface contributions should be negligible in this case. The primary source of error is attributed to a nonuniform geometry (axial dimension deviations and deviations from rectangular cross-sections, e.g., trapezoid). Minimal and maximal values of possible geometry deviations are used for modelling elastic modulus variations. These cross-section errors are detailed in Table S4 and Table S5, along with Figure S4, available in Supporting Information File 1. These error simulations of the geometry uncertainty indicate that a ±25 nm width error (SEM method) and ±15 nm height error (AFM method) for NWs with 100 nm width/height corresponds to the observed scattering in elastic modulus values in Figure 4.

Finally, point defects, such as oxygen vacancies, can increase the average bond length and thus result in a reduction in the elastic modulus [31–33]. For example, Wang et al. [34] showed that ZnO NWs with a higher density of oxygen vacancies, inferred from photoluminescence measurements, exhibited significantly (up to 20%) lower Young’s modulus. Wang et al. [35] measured a lower (up to 16%) elastic modulus for Al_2_O_3_ NBs in comparison to the theoretical value and attributed this difference to oxygen vacancies within NBs. As per our previous study [36], our as-grown Ga_2_O_3_ NWs exhibit a strong photoluminescence band related to oxygen vacancies, indicating a high concentration of such point defects. This could then partially be responsible for their lower elastic modulus values in comparison to that of the bulk material. Furthermore, planar structural defects, such as stacking faults, can also influence the mechanical properties of NWs. However, reports are showing that stacking faults can either decrease or increase Young’s modulus [31,37]. The decrease of Young’s modulus of NWs in comparison to that of bulk has been reported to be even as high as three times in WO_3_ NWs [25], four to five times in boron NBs [38], and up to 10 times in ZnO NBs [22]. This drastic difference from the bulk value is typically ascribed to a growth-direction-dependent concentration of stacking faults and point defects in NWs and NBs, which is correlated to the nanostructure cross-section aspect ratio (e.g., in ZnO nanostructures). Lower width-to-height ratio in NWs resulted in higher elastic modulus values, while NBs with higher width-to-height ratios showed a significant decrease in elastic modulus [22].

Although the variation of the cross-section geometry and the presence of different growth directions, related to the low symmetry of the monoclinic Ga_2_O_3_ phase and thus anisotropy in mechanical properties, are the most probable causes of the significant data scattering of the measured elastic moduli, the reason for the considerable decrease in comparison to the bulk value remains unclear. Our previous study of the as-grown Ga_2_O_3_ NW photoluminescence [36] implied a high concentration of oxygen vacancies, which could partially be responsible for the lower elastic modulus values. The TEM studies performed on a few selected NWs did not indicate the presence of stacking faults, which could contribute to higher elasticity. However, in order to gain full insight into the various factors affecting the elastic properties of Ga_2_O_3_ NWs, a further study should be performed on individual nanostructures with advanced combinatory methods, such as in-situ TEM mechanical resonance, to precisely determine the geometrical parameters, crystalline orientation, and presence of planar defects.

## Conclusion

In this work, elastic moduli of individual β-Ga_2_O_3_ NWs have been experimentally measured by two techniques: three-point bending and mechanical resonance. The obtained mean values are 34.5 GPa (standard deviation ±24.9) and 75.8 GPa (standard deviation ±47.6) from resonance and three-point bending methods, respectively. The measurements exhibited significant scattering, which was attributed to the variation of the cross-section geometry and the presence of different growth directions, related to the low symmetry of the monoclinic Ga_2_O_3_ phase and thus anisotropy in mechanical properties. This work demonstrates the need for finely controllable β-Ga_2_O_3_ NW synthesis methods and detailed post-examination of their mechanical properties before considering their application in future nanoscale devices.

## Experimental

### Materials

Ga_2_O_3_ NWs were synthesized using atmospheric pressure chemical vapour transport in a horizontal quartz tube reactor (18 mm inner diameter) according to the method reported in [2]. The process involved loading a ceramic boat with 0.15 g of Ga_2_O_3_ powder (99.99%, Alfa Aesar) at the centre of the quartz tube. Oxidised silicon wafers SiO_2_/Si (100) coated with Au nanoparticles (NPs, 100 nm of diameter, water suspension, Alfa Aesar) were positioned in a lower-temperature region 10 cm away from the furnace centre. Au NPs served as catalysts for the vapour–liquid–solid (VLS) growth mechanism. The reactor was heated to 1010 °C (high-temperature zone) under a carrier gas mixture of Ar/H_2_ 5%, maintaining this temperature and flow for 30 min to allow NW growth. Subsequently, the reactor was naturally cooled to room temperature. Ga_2_O_3_ NWs, up to 100 μm in length, grew on SiO_2_/Si substrates downstream in the low-temperature zone maintained around 850–900 °C.

### Characterisation

The morphology of as-grown NWs was examined using SEM (Helios 5 UX DualBeam). The measurements were carried out at an acceleration voltage of 5 keV and a beam current of 25 pA. Transmission electron microscopy (Tecnai GF20, FEI) at an accelerating voltage of 200 kV provided information on the crystalline structure of NWs. Fast Fourier transformation was performed on the obtained TEM images to determine crystalline orientations. The structure was also analysed using XRD on a Rigaku MiniFlex 600 X-ray powder diffractometer. The measurements were conducted in Bragg–Brentano θ/2θ geometry, utilising a 600 W Cu anode (Cu Kα radiation, λ = 1.5406 Å) X-ray tube.

In four steps, (100)Si wafers (Semiconductor wafer, Inc.) with 50 nm thermal oxide, were processed to create the patterned silicon substrates with grooves and inverted pyramids. First, the patterns were created in a photoresist on the wafer using conventional optical lithography. Next, the SiO_2_ was selectively removed using a buffered HF solution to replicate the resist pattern in the oxide layer. Then, the silicon was etched at 90 °C in a tetramethylammonium hydroxide (TMAH) solution to create etch pits. Finally, the remaining SiO_2_ was removed in HF. Two types of substrates were fabricated: one containing rectangular grooves with a depth of approximately 10 μm for resonance measurements, and the other containing inverted pyramid holes with depths ranging from hundreds of nm to a few µm three-point bending experiments.

The synthesized NWs were mechanically deposited onto the etched Si wafers. Nanowires, particularly those partially suspended over the groove with a free end, were selected for mechanical studies in SEM. Nanowires suspended over the inverted pyramid with both ends fixed were selected for three-point bending tests in AFM. The width and length of the selected NWs were determined using the Analyze–Measure function in the Fiji ImageJ2 software (version 1.53t) [39]. The selection of NW dimensions relied on the background contrast in SEM micrographs, ensuring an accurate determination of each start and endpoints of NWs.

### Mechanical resonance

**Figure 6:**
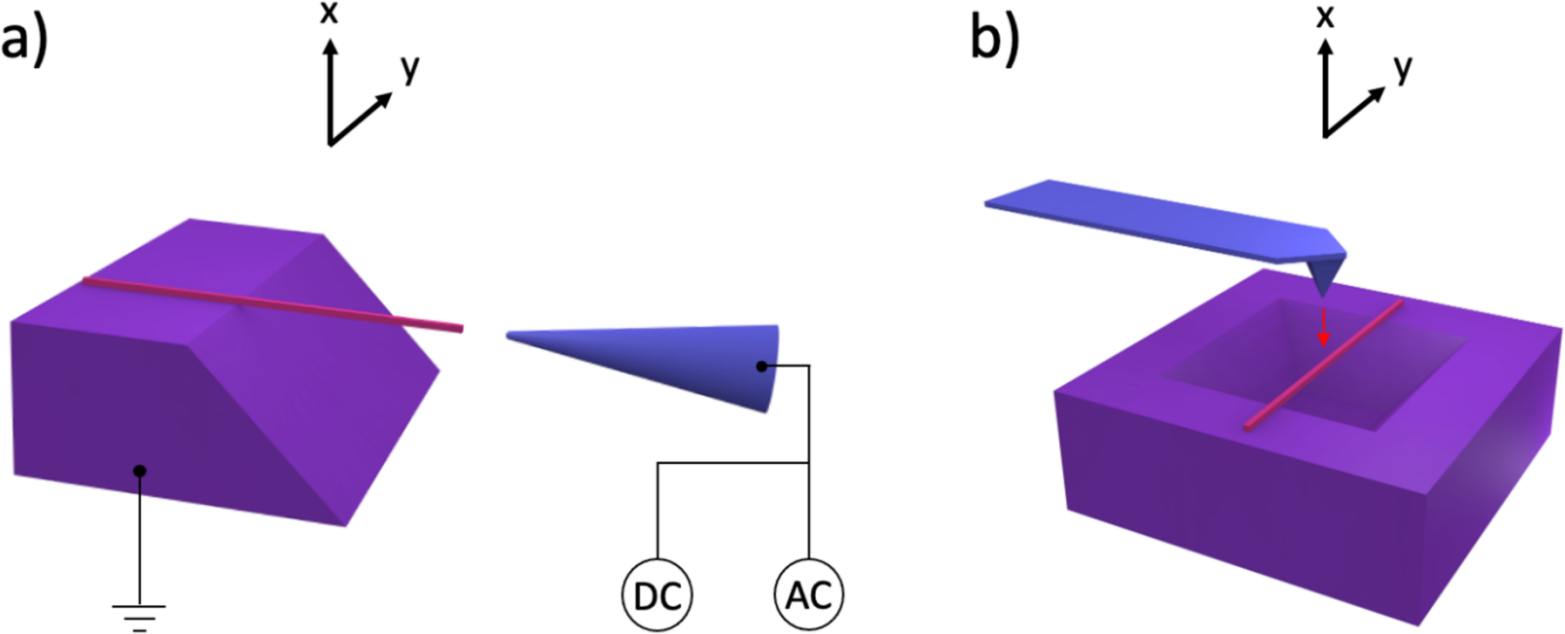
Schematic representation of the mechanical resonance and three-point bending tests: a) A NW suspended over the etched trench with one end fixed and a tungsten needle with applied potential for resonance measurements in SEM. Arrows “x” and “y” indicate NW resonance directions. b) A NW suspended over the inverted pyramid with both ends fixed for three-point bending measurements in AFM. The red arrow indicates the applied force direction.

Resonance measurements were executed using a micromanipulator (Kleindiek MM3A-EM) with a sharp tungsten probe. Resonance in the NWs was induced by applying a sinusoidal oscillating AC signal between the NW and the tungsten probe (electrode). The 4 V AC and 2 V DC excitation signal was generated by a waveform generator (RIGOL DG4162). For each NW, a resonance at its fundamental frequency was visually observed in SEM (Tescan Lyra, Figure 6a). If the applied frequency coincided with the frequency of the natural vibration, mechanical resonance was created due to an electric-induced charge on the oscillating tip of the NWs at the applied voltage frequency. In SEM, the selection of straight and horizontally oriented NWs was achieved by a focus test – when a NW remained in focus across its entire observed length, it was assumed to be in a single plane. This assumption suggests that the NW maintains a consistent horizontal alignment without deviating from a straight path throughout the observed region. Furthermore, to confirm the secure attachment of the NW to the substrate, seven NWs were “welded” to the substrate in SEM by Pt deposition using a gas injection system, and resonance frequencies were compared before and after the “welding” process (Table S2 in Supporting Information File 1). No significant difference was observed, indicating that the NWs were strongly fixed on the Si substrate and did not require any additional anchoring.

The elastic modulus is then calculated from observed resonance frequencies of NWs as follows [40]:


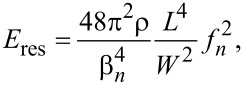


where *E*_res_ (of y-axis) is the elastic modulus, *β_i_* is a constant for the *i*-th harmonic (for the first harmonic (*i* = 1), β_1_ ≈ 1.875), *W* is the width of the NW, ρ is the density of the bulk Ga_2_O_3_, *ν* is the NW resonance frequency, and *L* is the length of the NW.

### Three-point bending tests

Three-point bending tests were performed by AFM (Dimension Edge, Bruker) using noncontact mode cantilevers with nominal stiffness of 42 N/m (NCHR-50, Nanosensors, Figure 6b). The NW lengths and widths for three-point bending were measured by SEM (Nova NanosEM 450, FEI). For every measured NW, many force–displacement curves were obtained. The images were analysed using the Gwyddion software (version 2.63).

Three-point bending is known as one of the most reliable methods for determining the elastic modulus (*E*_tpb_) of NWs and is calculated according to classical beam theory as follows:


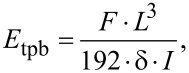


where *F* is the applied force, *L* is the suspended length, δ is the displacement of the NW, and *I* is the second area moment of inertia [41]. The second area moment of inertia for a rectangular beam is given by:


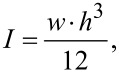


where *w* is the width of the NW and *h* is the height of the NW.

## Supporting Information

The Supporting Information contains details of values of mechanical resonance experiments in SEM and AFM, and profile measurements of NW in AFM and SEM images of Ga_2_O_3_ NWs with different cross-sections.

File 1 Additional results.

## Data Availability

All data that supports the findings of this study is available in the published article and/or the supporting information to this article.
