# Supplementary material for: Elastic modulus of β-Ga2O3 nanowires measured by resonance and three-point bending techniques
Source: Beilstein J Nanotechnol. 2024 Jun 18;15:704–12. doi: 10.3762/bjnano.15.58 (PMC11196948; doi:10.3762/bjnano.15.58)
Supplement: File 1 — Additional results. [file Beilstein_J_Nanotechnol-15-704-s001.pdf]

BEILSTEIN JOURNAL OF NANOTECHNOLOGY

Supporting Information

for

Elastic modulus of β-Ga_2O_3 nanowires measured by resonance and three-point bending techniques

Annamarija Trausa, Sven Oras, Sergei Vlassov, Mikk Antsov, Tauno Tiirats, Andreas Kyritsakis, Boris Polyakov and Edgars Butanovs

Beilstein J. Nanotechnol. **2024,** *15,* 704–712. doi:10.3762/bjnano.15.58

Additional results

License and Terms: This is a supporting information file under the terms of the Creative Commons Attribution License (https://creativecommons.org/licenses/by/4.0). Please note that the reuse, redistribution and reproduction in particular requires that the author(s) and source are credited and that individual graphics may be subject to special legal provisions.
The license is subject to the *Beilstein Journal of Nanotechnology* terms and conditions: (https://www.beilstein-journals.org/bjnano/terms)

Table S1 displays values obtained from mechanical resonance experiments conducted in a scanning electron microscope (SEM) for β-Ga_2O_3 nanowires (NWs). It includes dimensions of the NWs, resonance frequencies, and the corresponding calculated elastic modulus values.

Table S1: Mechanical resonance experiments in SEM.

	**Length (μm)**	**Width (nm)**	**Resonance frequency (kHz) (n=1)**	**Elastic modulus (GPa)**
1	13.578	50.572	132.20	**57.3**
2	10.100	80.650	149.62	**8.8**
3	6.016	77.397	669.46	**24.2**
4	14.045	103.432	126.26	**14.3**
5	13.230	102.795	126.75	**11.5**
6	26.900	70.520	32.56	**27.6**
7	8.845	78.302	263.13	**17.1**
8	15.384	182.415	283.01	**33.3**
9	16.570	56.310	90.08	**47.6**
10	12.618	81.660	196.11	**36.1**
11	8.587	76.840	577.63	**75.8**
12	12.618	81.390	186.50	**32.9**
13	9.830	48.770	125.16	**15.2**
14	16.550	50.640	42.34	**12.9**
15	6.720	60.000	769.84	**82.9**
16	15.900	89.760	178.60	**62.5**
17	16.090	165.410	213.11	**27.5**

18	12.210	63.280	72.00	**7.1**
19	22.280	103.590	110.73	**69.5**
20	15.950	103.330	240.20	**86.3**
21	7.750	89.250	250.85	**7.0**
22	19.560	101.320	92.58	**30.2**
23	16.450	118.870	208.79	**55.8**
24	18.500	122.780	46.82	**4.2**
25	8.670	91.270	389.40	**25.4**
26	11.440	75.900	180.72	**24.0**

Table S2 presents data derived from mechanical resonance experiments performed in SEM for β-Ga_2O_3 NWs. The table distinguishes between two sets of experiments: those where NWs are welded with Pt in SEM and those that are not. The values include dimensions of NWs and resonance frequencies, providing a comparative analysis of the mechanical properties of β-Ga_2O_3 nanowires with and without Pt welding in the SEM environment.

Table S2: Mechanical resonance experiments in SEM with and without Pt welding.

	**Length (μm)**	**Width (nm)**	**Resonance frequency (kHz) (n=1) before welding**	**Resonance frequency (kHz) (n=1) after welding**
1	6.016	77.397	669.46	690.53
2	13.230	102.795	126.75	129.98
3	8.845	78.302	263.13	265.29

4	15.384	182.415	283.01	284.30
5	12.618	81.660	196.11	191.00
6	7.750	89.250	250.85	264.60
7	19.560	101.320	92.58	94.90

In Table S3, the nanowire dimensions (length, width and height) are included providing a detailed characterization of the physical attributes of the β-Ga_2O_3 nanowires. The corresponding average of elastic modulus values, determined through three-point bending tests in an atomic force microscope (AFM) are shown as well as. The moment of inertia for each measured NW is also calculated and included.

Table S3: Three-point bending experiments in AFM.

	**Length (nm)**	**Width (nm)**	**Height (nm)**	**Moment of inertia (nm^4)**	**Average elastic modulus (GPa)**
1	2092.0	114.00	34.29	95517	128.70
2	2041.0	94.50	22.08	78265	104.60
3	2431.0	50.90	67.17	2514053	43.56
4	1248.5	44.93	54.00	730586	26.65
5	2379.5	121.37	82.83	4182029	63.96
6	1951.0	120.23	27.83	30546	143.88
7	1780.0	83.67	79.17	4281816	12.93
8	1486.0	68.60	47.33	606239	82.51

Figure S1 presents SEM images of Ga_2O_3 NWs with distinct cross-sections, notably a trapezoid (a) and rectangular shapes (b, c). These images illustrate the nonuniformity in the cross-sectional geometry of the NWs.

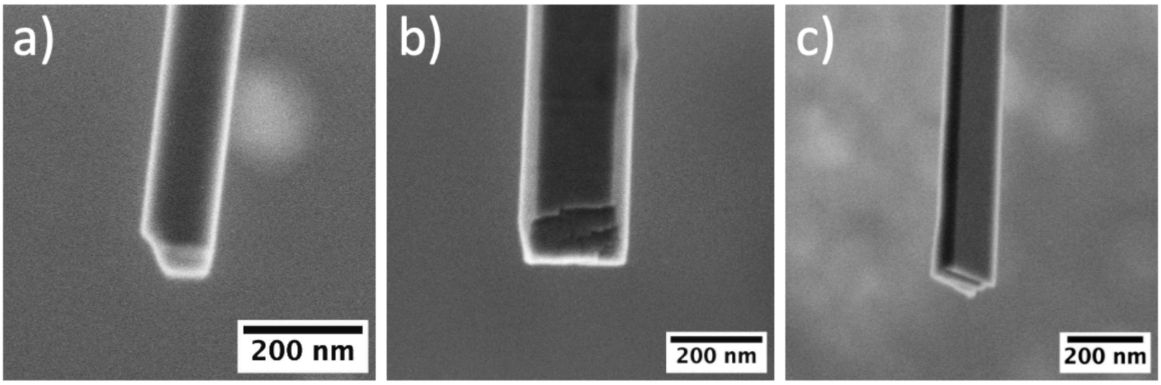

Figure S1: SEM images of Ga_2O_3 NWs with different cross-sections: a) trapezoid; b) and c) rectangular.

Figure S2 provides a schematic representation of a β-Ga_2O_3 NW positioned over an etched inverted pyramid on a Si substrate. The figure includes profile measurements obtained through AFM for a single NW. Notably, the height profile of the specific nanowire exhibits a variation, starting slightly above 20 nm at one end and increasing to 80 nm at the other end.

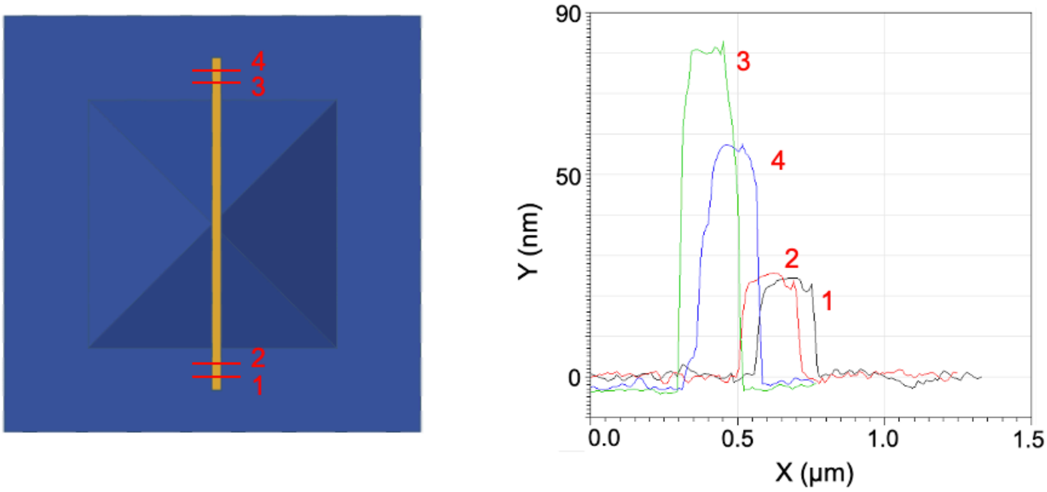

Figure S2: Height profiles of Ga_2O_3 NW.

Figure S3 displays the histogram of elastic modulus values obtained using two methods: the resonance method and the three-point bending method showing the frequency of elastic modulus values.

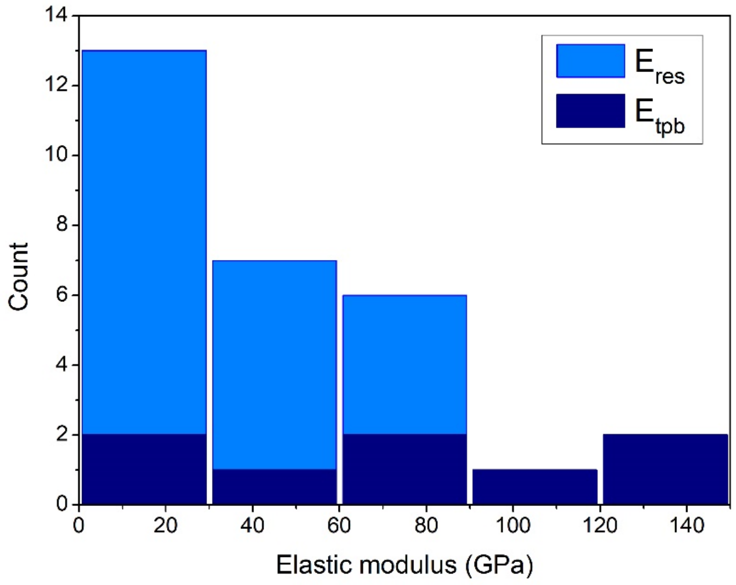

Figure S3: Histogram of elastic modulus values.

Table S4: Cross-section error in resonance experiments in SEM.

	**50 nm width**		**100 nm width**		**150 nm width**	
**NW width variation (nm)**	**Elastic modulus from minimal width (GPa)**	**Elastic modulus from maximal width (GPa)**	**Elastic modulus from minimal width (GPa)**	**Elastic modulus from maximal width (GPa)**	**Elastic modulus from minimal width (GPa)**	**Elastic modulus from maximal width (GPa)**
0	34.7	34.7	34.7	34.7	34.7	34.7
± 1	36.2	33.4	35.5	34.1	35.2	34.3
± 5	42.9	28.7	38.5	31.5	37.2	32.5
± 10	54.3	24.1	42.9	28.7	39.9	30.5
± 20	96.5	17.7	54.3	24.1	46.3	27.1
± 30	217.2	13.6	70.9	20.6	54.3	24.1

Table S5: Cross-section error in three-point bending experiments in AFM.

	**50 nm height**		**100 nm height**		**150 nm height**	
**NW width variation (nm)**	**Elastic modulus from minimal height (GPa)**	**Elastic modulus from maximal height (GPa)**	**Elastic modulus from minimal height (GPa)**	**Elastic modulus from maximal height (GPa)**	**Elastic modulus from minimal height (GPa)**	**Elastic modulus from maximal height (GPa)**
0	76.0	76.0	76.0	76.0	76.0	76.0
± 1	82.4	70.2	79.1	73.0	78.1	74.1
± 5	115.8	51.9	93.3	62.5	87.1	66.7
± 10	185.5	36.7	115.8	51.9	100.2	58.7
± 20	586.4	19.8	185.5	36.7	134.8	46.1
± 30	2968.8	11.6	316.5	26.6	185.7	36.7

In Table S4 and Table S5, minimal and maximal values of variations of geometry included in the elastic modulus calculation are shown. As depicted in Figure S2, NW profiles exhibit a nonconstant geometry, fluctuating within a 60 nm range, as illustrated in Figure S2. This variability, such as height ranging from 20 to 80 nm, poses challenges in accurately determining the geometry of the NW within the whole length of the NW. Despite the precision of both SEM and AFM measurements, our analysis suggests that the primary source of error stems from the nonconstant cross-sectional geometry of the NW. Variations of elastic modulus depending on variations of width in SEM resonance experiments are shown in Figure S4(a–c) and for variations of height in AFM three-point bending experiments are shown in Figure S4(d–f).

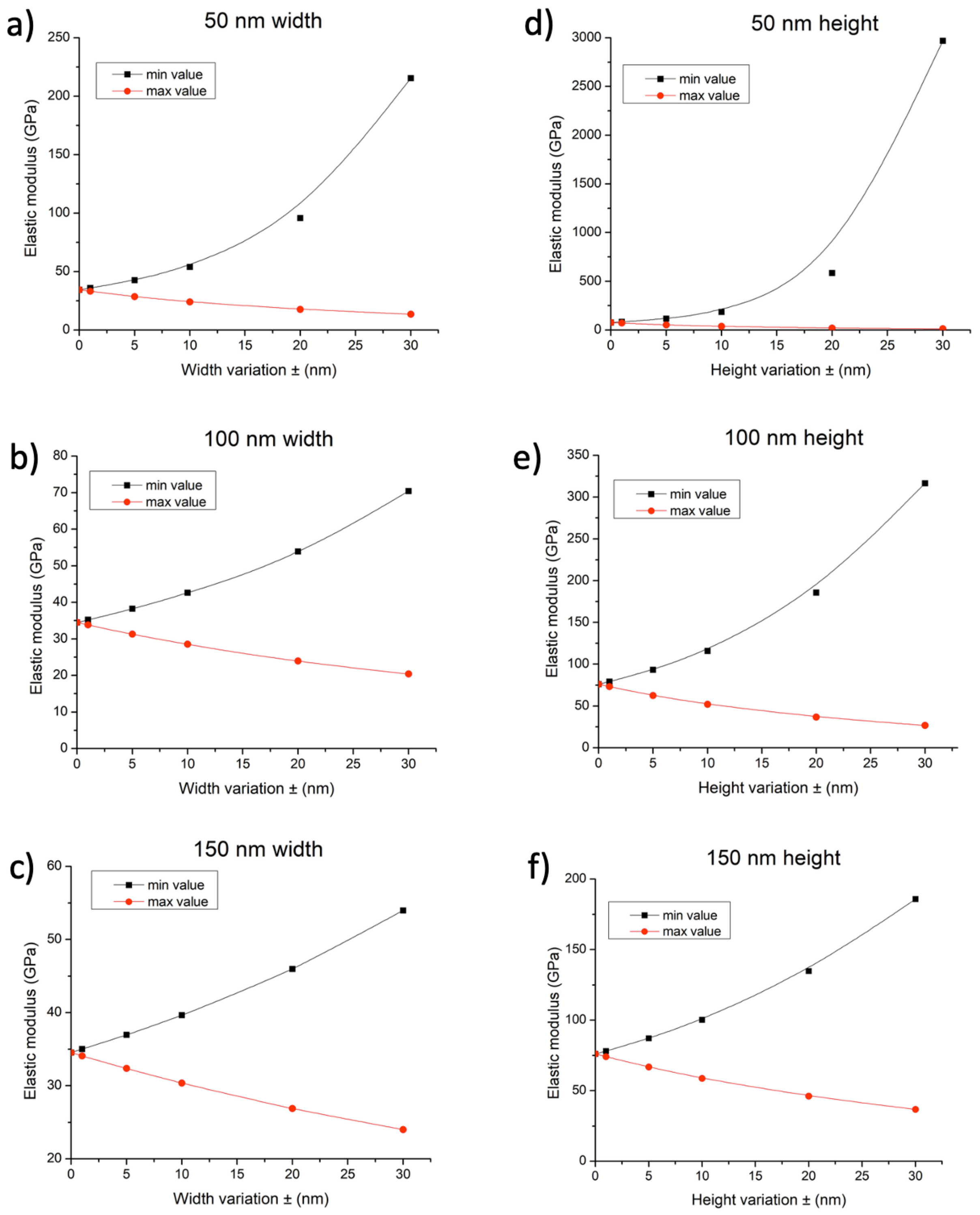

Figure S4: Graphs representing cross-section error. Variations of width in SEM resonance experiments are shown in (a–c), and for variations of height in AFM three-point bending experiments are shown in (d–f). Elastic modulus values, derived from the minimal and maximal values of width and height, are labelled as "min value" and "max value", respectively. Lines in graphs are drawn to guide the eye.
